# Nutritional Status of School Going Adolescent Girls in Awash Town, Afar Region, Ethiopia

**DOI:** 10.1155/2020/7367139

**Published:** 2020-02-21

**Authors:** Molla Kahssay, Lidia Mohamed, Abel Gebre

**Affiliations:** ^1^Department of Public Health, Samara University, Semera, Afar, Ethiopia; ^2^Afar Regional Health Bureau, Semera, Afar, Ethiopia

## Abstract

**Background:**

Adolescence is an essential stage in the human life cycle, a transition period between childhood and adulthood that is characterized by rapid growth spurt in which nutritional requirement is high. Adolescents are risk groups for malnutrition, but they are not part of a target in many intervention strategies. Hence, this study was aimed at assessing nutritional status of adolescent girls and its associated factors.

**Methods:**

Institutional based cross-sectional study design was employed among randomly selected 348 school going adolescent girls. Data were entered into Epi Info and transported to SPSS version 20 for further analysis. Binary logistic regression analysis was used to identify predicators of nutritional status of adolescent girls at *p* value <0.05 and 95% confidence level.

**Results:**

This study revealed that 22.9% and 8.82% of school adolescent girls were stunted and thin, respectively. Being at early adolescent age (14-15 years) [AOR = 1.4, 95% CI (1.04–4.28)], ownership of phone [AOR = 3.3, 95% CI (1.55–7.02)], and dietary diversity score of <4 food groups [AOR = 2.2, 95% CI (1.4–4.54)] were some of the potential predictors of stunting. Similarly, dietary diversity score of <4 food groups [AOR = 1.8, 95% CI (1.14–4.38)] and low food consumption [AOR = 3, 95% CI (1.15–7.90)] were some of the potential predictors of thinness. *Conclusion and Recommendation*. The prevalence of both stunting and thinness is a public health problem in the study area. Early adolescent age (10–14 years), ownership of phone, and dietary diversity score of <4 food groups were independent predictors of stunting. Dietary diversity score of <4 food groups and eating less than usual were independent predictors of thinness. An integrated nutritional intervention and health related services that meet the needs of adolescent girls in the school community have to be established and strengthened. Since adolescent age is period of growth and development in which growth spurt and nutritional requirement are high, adolescents should be provided with enough meals and diversified foods.

## 1. Background

Adolescence is a pivotal period of development which represents the age of 10–19 years. Adolescence is a tap root growth and development life stage which has implications for future nutritional status and food consumption habits. Adolescent girls need to have good quantity and quality nutrients to cope with this rapid growth and other health risks which increase nutritional demand [1]. Adolescents account for about one-fourth of the total world's population, and the majority of them live in developing countries. Greater than one-third (38.6%) of Ethiopian population were found in this age group making Ethiopia the third country in the world [2]. Adolescence is an essential stage in the human life cycle, a transition period between childhood and adulthood that is characterized by rapid growth spurt [3, 4].

Adolescent girls thus need to be adequately nourished to ensure their own optimal growth and maturation, in preparation for their future reproductive capacity during this crucial period. The physical and psychosocial changes occurring during childhood and adolescence make this age group more vulnerable to health and nutrition concerns compared to others. Because of pubertal growth and menarche during this period, the adolescents are requiring the highest quantity and quality nutrients. Consequently, if those requirements and quality of nutrients for adolescents are not met, malnutrition happens, which influences growth, development, and health of adolescents [5, 6].

In Africa the prevalence of undernutrition among adolescents was found to be higher in the eastern part of the continent [7, 8]. In Ethiopia the prevalence of undernutrition was found to be high [7, 9]. Undernutrition is a major public health problem in the majority of Ethiopian communities including urban and rural adolescents [10, 11]. In most developing countries, nutrition initiatives have been focusing on children and women, thus neglecting adolescent girls. Addressing the nutrition needs of adolescents could be an important step towards breaking the vicious cycle of intergenerational malnutrition, chronic diseases, and poverty. Malnutrition during adolescence can have lasting consequences on an adolescent's cognitive development, resulting in decreased learning ability, poor concentration, and impaired school performance [12].

Malnutrition is associated with significant morbidity and mortality and affects the reproductive outcome in adolescent girls. Moreover, undernourished adolescents tend to be ultimately malnourished adults, give birth to small babies, and transmit undernutrition to future generation [9]. The health consequences on adolescent girls have been identified to be high if they are short and underweight and transmit malnutrition to next generation because of competing growth of mother [13, 14]. Despite the emergence of a number of advancements in areas of health and nutrition services in developing countries including Ethiopia, nutritional status of adolescents is not yet commonly included in health and nutrition surveys and an up-to-date overview of their nutritional status across the world is not available [15]. Within the past few years, emerging research on adolescent health in sub-Saharan Africa describes a high prevalence of malnutrition, especially among girls [16]. Attaining health for all people at every stage of their life especially for the adolescents, the so called next generation, is impossible in the presence of malnutrition [12, 17, 18].

In Ethiopia, so far, limited studies have been conducted with regard to adolescent nutrition, and there was no similar study conducted in pastoral community. Hence, this study aimed to assess nutritional status of school going adolescent girls and its associated factors in Awash town, Afar Region, Ethiopia.

## 2. Methods

### 2.1. Study Area

The study was conducted in Afar Region, Ethiopia. Afar is located in the eastern part of Ethiopia. The Afar Regional State consists of 9 administrative zones, 32 woredas and 5 urban administrations/towns. The area is characterized by a harsh climate with temperatures up to 40°C, highly variable average precipitation between 5 and 600 mm annually, and recurrent droughts and floods; under these conditions mobile pastoralist is the dominant type of land use due to its high adaptive capacity. As of 2018, Awash town has one primary and one secondary and preparatory school which are owned by the government.

### 2.2. Study Period

The study was conducted from January to February 2018.

### 2.3. Study Design

Facility based cross-sectional study was employed.

### 2.4. Source Population

All adolescent girls found in all schools of Awash town constituted the source population of this study.

### 2.5. Study Population

Adolescent girls attending the selected Awash primary, secondary, and preparatory schools constituted the study population.

### 2.6. Eligibility Criteria

All adolescent girls aged 10 to 19 years attending the selected schools during the study period were part of the study, and adolescent girls with obvious physical deformities for anthropometric measurements and/or who were seriously ill to be interviewed were excluded from the study.

### 2.7. Sample Size Determination

The sample size was determined using single population proportion formula, considering 95% confidence interval, 80% power, and 5% marginal error, with the proportion of adolescents who were stunted being 31.5% [19].

(1) $$\begin{array}{c} n=\frac{\left({\left(z_{\alpha /2}\right)}^{2}*P\left(1-P\right)\right)}{\left(d^{2}\right)},\\ n=\frac{\left({\left(1.96\right)}^{2}\left(0.315*0.685\right)\right)}{{\left(0.05\right)}^{2}},\\ n=331. \end{array}$$

Adding 5% nonresponse rate, the final sample size was 348 subjects.

### 2.8. Sampling Procedures

**Figure 1:**
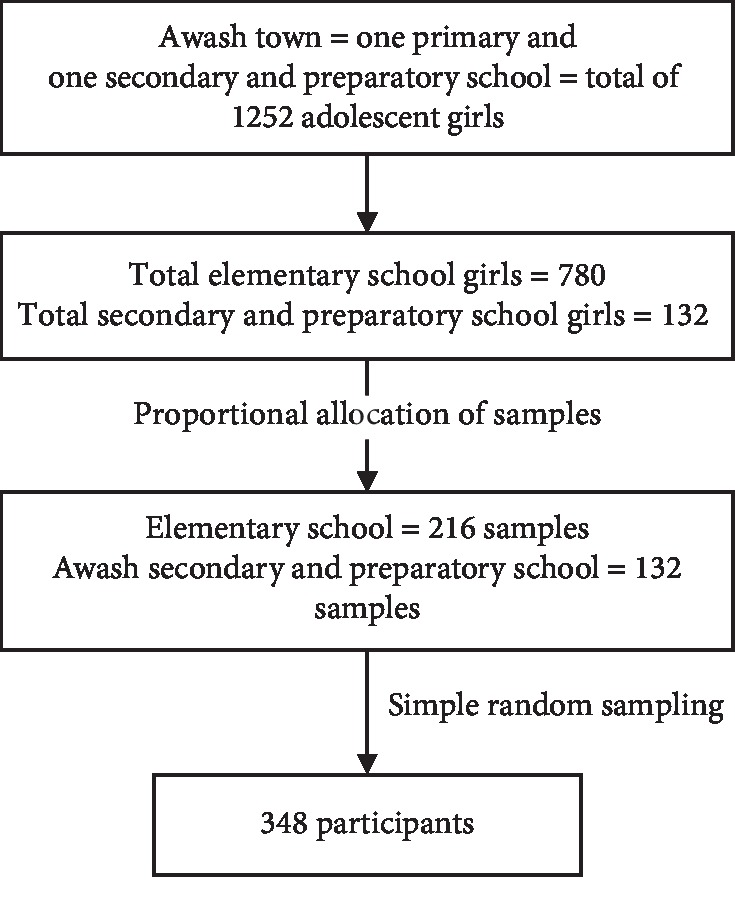
Schematic representation of sampling procedure of school adolescent girls, Awash town, Afar, Ethiopia.

According to Awash Town Education Bureau report, the town had one primary school and one secondary and preparatory school. The total adolescent girls in the schools were 1252. Elementary, secondary, and preparatory school adolescent girls were taken using population proportion to size allocation based on the number of adolescent girls in each school. Finally, study participants were selected using a simple random sampling technique from sampling frame that was made from all school registers obtained (Figure 1).

### 2.9. Operational Definition of Terms

The definitions of terms were as follows:

  Adolescents: individuals in the age group of 10–19 years
  Thinness: BMI-for-age < −2 *Z* scores of the 2007 WHO reference
  Stunting: height-for-age < −2 *Z* scores of the 2007 WHO reference
  Poor dietary diversity: adolescent girls with dietary diversity score of <4 food groups
  Good dietary diversity: adolescent girls with dietary diversity score of ≥4 food groups

### 2.10. Data Collection Process

**Figure 2:**
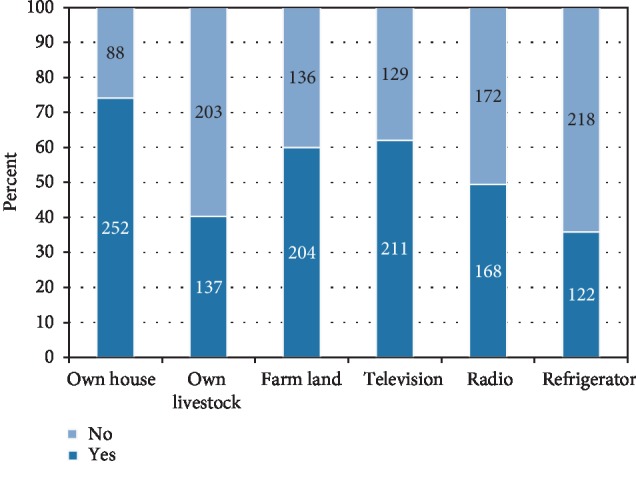
Household asset possession of school going adolescent girls, Awash town, 2018: (*n* = 340).

**Figure 3:**
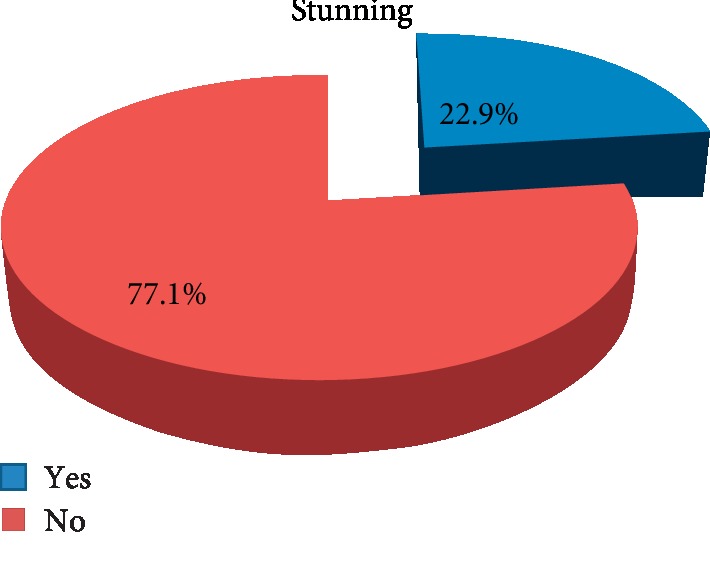
Prevalence of stunting among school going adolescent girls, Awash town, 2018: (*n* = 340).

**Figure 4:**
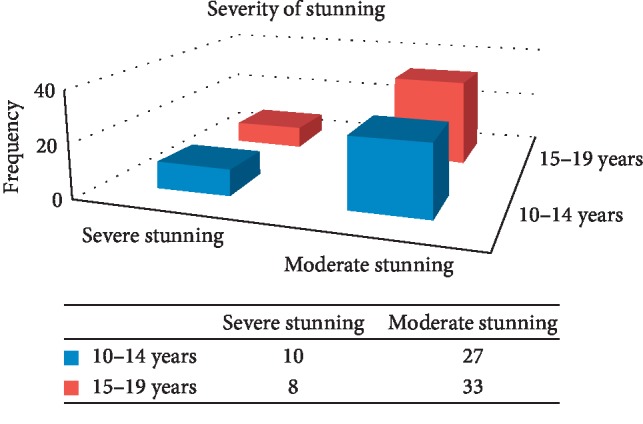
Prevalence and degree of stunting by age among school going adolescent girls, Awash town, 2018: (*n* = 340).

**Figure 5:**
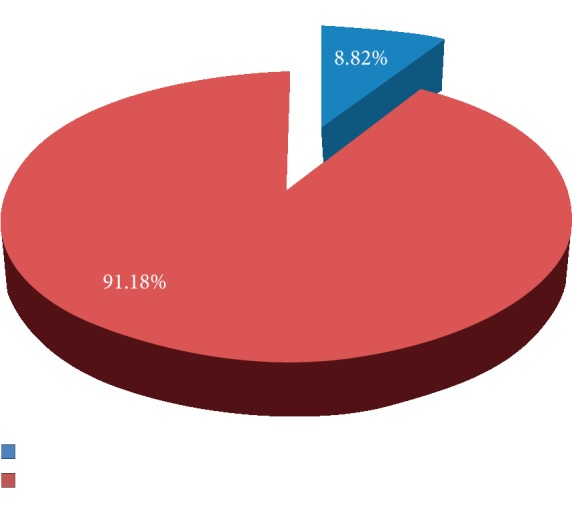
Prevalence of thinness among school going adolescent girls, Awash town, June, 2018: (*n* = 340).

**Figure 6:**
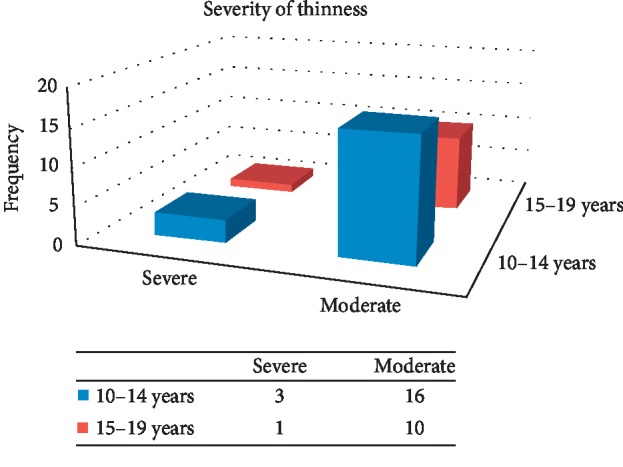
Prevalence and degree of thinness by age among school going adolescent girls, Awash town, 2018: (*n* = 340).

Data was collected using an interviewer who administered structured questionnaire, taking anthropometric measurements (weight, height) of the study subjects. The questionnaire was adapted from previous studies after a thorough review of different studies. Stadiometers with a sliding headpiece attached to digital weight scale were used to measure height and weight, respectively. During anthropometric measurements, weight was measured to the nearest 0.1 kg and height to the nearest 0.1 cm in standing position. Periodic calibration of the instruments was made by placing standard calibration on the scale. Anthropometric measurements were converted to height-for-age and BMI-for-age *Z* scores by using AnthroPlus software. Girls with height-for-age below −2 *Z* scores and BMI-for-age below −2 *Z* scores of the 2007 WHO reference population were classified as stunted and thin, respectively (Figures 2–6).

### 2.11. Data Quality and Control

The English version of the structured questionnaire was translated into local language “Afaraf” and again back-translated to English by another translator to assure the consistency of the questions. Data collectors and supervisors were trained for three days on standardization of the anthropometric tools. All anthropometric tools were tested to ensure that each tool produces the same measure of a standard object. To test for accuracy, the scales were checked by placing items of known weight on them after every 10 measurements. The scale was regularly checked and adjusted to zero after each measurement. Pretest was done in 5% of the total sample before the actual survey out of the study setting to ensure clarity, ordering, consistency, and acceptance of the questionnaire. To improve quality of the data, data collectors were closely supervised, and each completed questionnaire was checked to ascertain that all questions were properly filled and corrected.

### 2.12. Data Processing and Analysis

After data collection, the data was cleaned, coded, entered into Epi Info, and then exported to SPSS version 20 for further analysis. Anthropometric indices were calculated by using WHO AnthroPlus software. Descriptive statistics was used to show the prevalence of stunting and thinness and other sociodemographic characteristics. All variables having a *p* value of <0.25 in the univariable analysis were candidates for multivariable logistic regression model. In the multivariable analysis, variables with *p* value <0.05 were taken as significant predictors for stunting and thinness at a 95% confidence interval.

### 2.13. Ethical Consideration

Ethical approval was obtained from Research and Ethics Review Committee at Samara University. Support letter was also obtained from respective regional and district education offices as well as from all the selected schools in Awash town. After permission was obtained from school administration, the parental consent was obtained the day prior to the data collection. After the written consent was obtained from parents of study subjects, assent was obtained by explaining the purpose and the importance of the study to the adolescent girls with standard assurance of confidentiality.

## 3. Results

### 3.1. Sociodemographic and Economic Characteristics of Study Participants

A total of 340 adolescent girls participated in the study with response rate of 97.7%. More than half (53%) of the study participants were from Awash secondary and preparatory school. Of the total adolescent girls, 135 (39.7%) were aged 10–14 years (early adolescence) and 205 (60.7%) were in the age category of 15–19 years (late adolescence). Regarding religion, 162 (47.6%), 122 (35.9%), and 55 (16.2%) of the participants were Muslims, Orthodox Christians, and Protestants, respectively (Table 1).

### 3.2. Dietary Intake Characteristics of Study Participants

Purchased and own products were source of staple foods for 201 (59.1%) and 101 (29.7%) of the study participants. Teff was the most cited staple food for 294 (86.5%) of the study participants whereas wheat was the least listed staple food for 20 (5.9%) of the study participants. According to study participants 24 hour recall report, the number of food groups consumed was computed. Almost half (49%) of the study participants had consumed foods from <4 food groups. The mean consumption of diversified food was 2.81 ± (SD = 0.501) food groups (Table 2).

### 3.3. Life Style and Behavior Characteristics of Study Participants

One hundred thirty-six (40%) of the study participants claimed that they had regular physical activities. Khat chewing was practiced in 86 (25.3%) of the study subjects. Cigarette smoking and alcohol consumption were practiced in 2 (0.6%) and 31 (9.1%) of families of study participants, respectively. Based on self-estimation of body size, 126 (37.1%), 58 (17.1%), and 99 (29.1%) of the participants considered themselves as thin, medium, and very fat, respectively. Of the total study participants 85 (25%) and 47 (13.8%) participants attempted to gain weight and to lose weight, respectively.

### 3.4. Sanitation and Hygiene Characteristics of Study Participants

All selected schools had latrine but none of them had hand washing facilities. The majority of study participants 262 (77.2%) had latrine in their home, of which only 128 (37.6%) had hand washing facilities. The most common type of latrine was pit latrine without slab 153 (45%) followed by pit latrine with slab 57 (16.8%). Most of the study participants were obtaining water from tap (99.4%). About 13 (4.5%) and 16 (4.7%) of the study subjects did not wash their hands before eating food and after using toilet, respectively.

### 3.5. Reproductive Characteristics of Study Participants

Among respondents about 232 (68.2%) of them claimed that they started menstruating whereas 108 (31.8%) of them did not start menstruating. Of the total respondents, 339 (99.7%) had no previous history of utilization of family planning methods.

### 3.6. Nutritional Status of Study Participants

#### 3.6.1. Stunting

This study revealed that 78 (22.9%) of school adolescent girls were stunted.

#### 3.6.2. Thinness

This study revealed that 30 (8.82%) of school adolescent girls were thin.

### 3.7. Factors Associated with Stunting

In univariable binary logistic regression analysis adolescent age, adolescent family size, source of food, dietary intake of adolescents, and family possession of phone were some of the variables with *p* value <0.25 and were candidates for multivariable analysis. In multivariable binary logistic regression analysis being at early adolescent age (10–14 years), ownership of phone and poor consumption of diversified foods (low DDS) were independent predictors of stunting.

Adolescents aged 10–14 years were 1.4 times more likely to be stunted compared to adolescents aged 15–19 years (AOR = 1.4, 95% CI (1.04–4.28)). Adolescents from families who did not possess phone were about three times more likely to be stunted compared to their counterparts (AOR = 3.3, 95% CI (1.55–7.02)). Regarding dietary intake, adolescents who had poor consumption of diversified foods (DDS < 4 food groups) were 2.2 times more likely to be stunted compared to those who had good consumption (DDS ≥ 4 food groups) (AOR = 2.2, 95% CI (1.4–4.54)). (Table 3).

### 3.8. Factors Associated with Thinness

In univariable binary logistic regression analysis, consumption of fruit and vegetables, reducing meal in household, and changing feeding habit were some of the variables with *p* value <0.25 and were candidates for multivariable analysis. In multivariable binary logistic regression analysis, poor consumption of diversified foods (DDS < 4 food groups) and eating less than usual were independent predictors of thinness. Adolescents who had poor consumption of diversified foods (DDS < 4 food groups) were 1.8 times more likely to be thin compared to their counterparts (AOR = 1.8, 95% CI (1.14–4.38)). Adolescents who did eat less because of household financial constraints were three time more likely to be thin than their counterparts (AOR = 3, 95% CI (1.15–7.90)) (Table 4).

## 4. Discussion

This study was conducted to determine nutritional status and associated factors among school adolescent girls in Awash town. The main nutritional problem which affects adolescents is undernutrition in terms of stunting and thinness. This study revealed a prevalence of thinness and stunting of 8.82% and 22.9%, respectively. The prevalence of stunting in this study was comparable with that in a study conducted in Eastern Arsi Zone which was 20.2% [20] and national nutrition survey report of Ethiopia 23% [21], but less than the finding in Amhara Region 31.5% [19]. The possible reason for this difference in prevalence could be a difference in socioeconomic status. However, the prevalence of stunting in this study was greater than studies conducted in Adama city, 15.6% [22], and Adwa town, Tigray, 12.2% [23]. The possible reason for this variation could be the difference in the study setting, as this study was conducted in urban adolescent girls, while the above studies were done in both urban and rural areas whereby health service utilization and factors which determine health are quite different.

The prevalence of thinness in this study was 8.8%. This result is similar to that in a study conducted in Northwest Ethiopia, Gondar town, which was 10.4% [24]. This study result is lower than the study results conducted at Amhara, 13.6% [19], and rural community of Aseko district, Eastern Arsi Zone, and Oromia region, 14.8% [20]. This variation might be related to the nature of the diet and lifestyle of the individual or may be attributed to poor socioeconomic conditions in the study settings.

Regarding factors associated with stunting, this study has found that adolescents aged 10–14years were 1.4 times more likely to be stunted compared to adolescents aged 15–19 years (AOR = 1.4, 95% CI (1.04–4.28)). This finding is consistent with some research findings [9, 19]. Those early adolescents are at the greatest gain in height as compared to late adolescents. Hence, failing to achieve their nutrient needs for this period will make early adolescents more susceptible for developing chronic malnutrition.

The findings of this study showed that adolescent girls from families who did not possess phone were about three times more likely to be stunted compared to their counterparts (AOR = 3.3, 95% CI (1.55–7.02)). Regarding dietary intake, adolescents who had poor consumption of diversified foods (DDS < 4 food groups) were 2.2 times more likely to be stunted compared to those who had good consumption (DDS ≥ 4 food groups) (AOR = 2.2, 95% CI (1.4–4.54)). This might be because dietary diversity is the proxy indicator of dietary habit and having poor dietary habit can lead to stunting.

According to this study finding, adolescent girls who did eat less because of household financial constraints were three time more likely to be thin than their counterparts (AOR = 3, 95% CI (1.15–7.90)). This finding is consistent with a study done in Amhara Region, Northwestern Ethiopia [19]. This might be explained by the fact that failing to achieve nutrient needs can be manifested by thinness. Adolescent girls who had poor consumption of diversified foods (DDS < 4 food groups) were 1.8 times more likely to be thin compared to their counterparts (AOR = 1.8, 95% CI (1.14–4.38)). This might be explained by the fact that poor dietary diversity is the proxy indicator of poor dietary habit which can lead to thinness.

### 4.1. Strengths of the Study

The strength of this study lies in its focus on adolescent girls, a group that is presently lacking attention. This study also used primary data source.

### 4.2. Utility of the Study

The present research has enormous social relevance and utility. This study provided information on the nutrition status of school adolescent girls and significant predictors of stunting and thinness. This could be used as a baseline data for other researchers and will provide evidence for administrators and policy makers to plan prevention strategies for undernutrition among adolescent girls.

### 4.3. Limitations of the Study

The primary limitation of this study is that it is cross-sectional. Because the exposure and outcome were assessed simultaneously, we cannot claim a causal relationship between the identified predictor variables and the outcome variable (undernutrition).

## 5. Conclusion and Recommendation

The findings of the study have shown that stunting and thinness were high among school adolescent girls. One out of four adolescents was stunted. Early adolescent age (10–14 years), ownership of phone, and poor consumption of diversified foods (DDS < 4 food groups) were independent predictors of stunting. Poor consumption of diversified foods (DDS < 4 food groups) and eating less than usual were independent predictors of thinness. An integrated nutritional intervention and health related services that meet the needs of adolescent girls in the school community have to be established and strengthened. Since adolescent age is period of growth and development in which growth spurt and nutritional requirement are high, adolescents should be provided with enough meals and diversified foods. Families as well as the whole community have to be aware of nutrition of adolescent girls through health extension workers and routine facilities service.

## Figures and Tables

**Table 1:** Sociodemographic and economic characteristics of school going adolescent girls, Awash town, 2018 (*n* = 340).

Variable	Category	Frequency	%
Age	10–14	135	39.7
	15–19	205	60.3

School grade	1–8	160	47
	9–12	180	53

Family size	<= 3	42	12.4
	4–5	94	27.6
	>5	204	60.0

Religion	Muslim	162	47.6
	Orthodox	122	35.9
	Protestant	56	16.5

Living	With parent	278	81.8
	Renting alone	9	2.6
	With relatives	53	15.6

Absence from school in a month	≥5 days	43	33.6
	<5 days	85	66.4

Work other than being student	Yes	46	13.5
	No	294	86.5

**Table 2:** Dietary intake characteristics of school going adolescent girls, Awash town, 2018 (*n* = 340).

Source of food	Own product	101	29.7%
	Market purchase	201	59.1%
	Gift	6	1.8%
	Exchange and barter	12	3.5%
	Humanitarian food aid	16	4.7%
	Loan	4	1.2%

Mainly used food staples in the area	Teff	294	86.5%
	Maize	11	3.2%
	Sorghum	15	4.4%
	Wheat	20	5.9%

Number of hunger episodes in last month	No episodes	15	4.4%
	One episode	5	1.5%
	Two episodes	35	10.3%
	Three episodes	71	20.9%
	Four and above episodes	214	62.9%

Diet diversity	Poor (<4 food groups)	167	49%
	Good (≥4 food groups)	173	51%

**Table 3:** Multivariable binary logistic regression analysis for determinants of stunting among school going adolescent girls of Awash town, 2018 (*n* = 340).

Variables	Stunting		COR (95%)	AOR (95% CI)
	Yes (%)	No (%)		
Age				
10–14	37 (47.4)	98(37.4)	1.51(1.12–5.43)	1.4(1.04–4.28)^*∗*^
15–19	41 (52.6)	164 (62.6)	1	

Family size				
< = 3 persons	7 (9.0)	35 (13.4)	1	
4–5 persons	18 (23.1)	76 (29.0)	1.18(.45–3.09)	1.31(0.48–3.57)
>5 persons	53(67.9)	151(57.6)	1.75(.74–4.19)	1.87(0.75–4.63)

Availability of phone				
Yes	63 (80.8)	240 (91.6)	1	.
No	15 (19.2)	22 (8.4)	2.56 (1.27–5.29)	3.3(1.55–7.02)^*∗*^

DDS				
Good	29(37.2)	144(55)	1	1
Poor	49(62.8)	118(45)	2.06(1.23, 5.64)	2.2(1.4, 4.54)^*∗*^

Fruits and vegetables intake				
Yes	39 (50.0%)	90 (34.4%)	1.91 (1.15–3.19)	1.72 (0.14–4.25)
No	39 (50.0%)	172 (65.6%)	1	

COR  crude odds ratio, AOR  adjusted odds ratio, CI  confidence interval, ^*∗*^significant at *p* < 0.05.

**Table 4:** Multivariable binary logistic regression analysis for determinants of thinness among school going adolescent girls of Awash town, 2018 (*n* = 340).

Variables	Thinness		COR (95%)	AOR (95% CI)
	Yes (%)	No (%)		
Fathers' occupation				
Pastoral	1(3.3%)	12(3.9%)	.85(.09–7.33)	.93(0.09–8.91)
Agro pastoral	3(10.0%)	36(11.6%)	.85(.22–3.33)	.83(0.17–4.15)
Merchant	9(30.0%)	86(27.7%)	1.070(.41–2.82)	1.49(0.47–4.72)
Daily laborer	8(26.7%)	84(27.1%)	.97(.36–2.64)	1.39(.42–4.64)
Government employee	9(30.0%)	92(29.7%)	1	1

Mothers' occupation				
Pastoral	2(6.7%)	19(6.1%)	1.09(.16–7.12)	1.97(0.23–16.46)
Agro pastoral	1(3.3%)	11(3.5%)	.94(.08–10.00)	3.53(0.24–52.54)
Merchant	9(30.0%)	93(30.0%)	1.0(.25–3.93)	1.68(0.35–8.11)
Daily laborer	5(16.7%)	64(20.6%)	.81(.18–3.57)	2.34(0.41–13.36)
Government employee	10(33.3%)	92(29.7%)	1.12(.29–4.35)	4.23(0.77–23.1)
Housewife	3(10.0%)	31(10.0%)	1	1

Feeding habit				
As usual	17(56.7%)	189 (61%)	1	
More than usual	12(40%)	81(26.1)	1.65 (.75–3.61)	1.77 (0.72–4.34)
Less than usual	1(3.3%)	40(12.9%)	.28 (.04–2.15)	.25 (0.03–2.01)

Eating less than usual				
Yes	23(76.7%)	157(50.6%)	3.2(1.34–7.68)	3.01(1.15–7.90) ^*∗*^
No	7(23.3%)	153(49.4%)	1	

DDS				
Good	14(47)	159(61)	1	1
Poor	16(53)	103(39)	1.76(1.22, 4.86)	1.8(1.14, 4.38)^*∗*^

Fruit and vegetable consumption				
Yes	19(63.3%)	110(35.5%)	1	
No	11(36.7%)	200(64.5%)	0.32(0.15–0.69)	0.28 (0.11–1.78)

Milk consumption				
Yes	2(6.7%)	36(11.6%)	1	
No	28(93.3%)	274(88.4%)	1.84(0.42–8.05)	0.29(0.06–1.48)

COR  crude odds ratio, AOR  adjusted odds ratio, CI  confidence interval, ^*∗*^significant at *p* < 0.05.

## Data Availability

All the results of this research were based on the use of primary data, and the data collection was performed prospectively. The datasets supporting the conclusions of the study are included in the article. Any additional data will be available from the corresponding author on reasonable request.

## Abbreviations

CSA: Central Statistical Agency
DDS: Dietary diversity score
EDHS: Ethiopia Demographic and Health Survey
FAO: Food and Agriculture Organization
ICRW: International Center for Research on Women
NHNES: National Health and Nutrition Examination Survey
SPSS: Statistical Package for the Social Sciences.
